# A Quite Rare Association: Levo-Malposition of the Great Arteries with Left Juxtaposition of the Atrial Appendages in “Double Outlet Right Ventricle"

**DOI:** 10.21470/1678-9741-2020-0220

**Published:** 2021

**Authors:** Betül Çınar, Erkut Öztürk, Okan Yıldız, Sertaç Haydın, Alper Güzeltaş

**Affiliations:** 1 Department of Pediatric Cardiology, Istanbul Mehmet Akif Ersoy Thoracic and Cardiovascular Surgery Center and Research Hospital, Istanbul, Turkey.; 2 Department of Pediatric Cardiovascular Surgery, Istanbul Mehmet Akif Ersoy Thoracic and Cardiovascular Surgery Center and Research Hospital, Istanbul, Turkey.

**Keywords:** Atrial Appendage, Heart Defects, Congenital, Double Outlet Right Ventricle, Child

## Abstract

Although many anatomical variations may be encountered in children with double outlet right ventricle, coexistence of levo-malposed great vessels and left juxtaposed atrial appendages is uncommonly observed. This case report underlines the rarity of this anatomical combination and its clinical significance along with the surgical management in an infant.

**Table:** 

Abbreviations, acronyms & symbols	
**Ao** **DORV** **LV** **PA** **RV** **S,D,L** **VSD**	**= Aorta** **= Double outlet right ventricle** **= Left ventricle** **= Pulmonary artery** **= Right ventricle** **= Situs solitus, D-loop ventricles, and L-position of the aorta** **= Ventricular septal defect**

## INTRODUCTION

Double outlet right ventricle (DORV) is a type of conotruncal deformity in close relationship with complex cardiac anomalies, which are bound together by a characteristic that both great arteries emerge predominantly from the right ventricle. In those patients, clinical presentation and treatment vary according to the spatial position of great arteries, localization of ventricular septal defect (VSD), and accompanying atrial juxtaposition ^[1,2]^. In this case, we aim to engage awareness to an unusual association of left juxtaposed atrial appendages in a rare subgroup of DORV patients with levo-malposed great arteries and a subaortic VSD.

## CASE REPORT

**Fig. 1:**
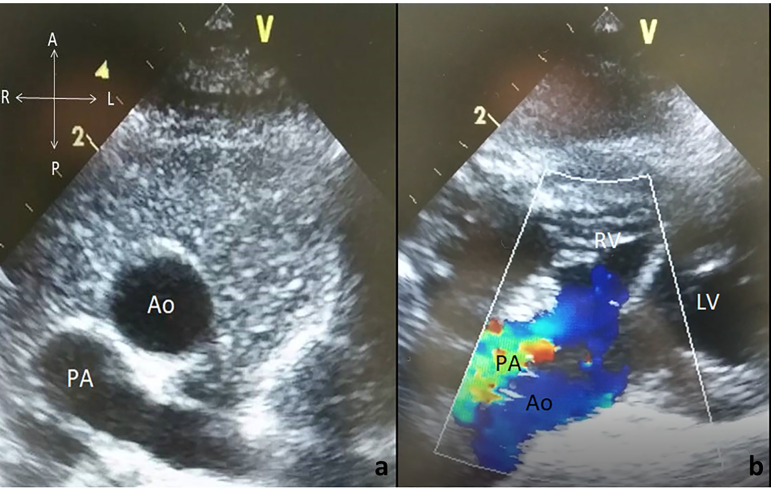
Echocardiographic two-dimensional image demonstrating the spatial relations of the great arteries in modified parasternal short axis view (a); color Doppler image showing that both great arteries are emerging from the right ventricle in modified subcostal view (b). Ao=aorta; LV=left ventricle; PA=pulmonary artery; RV=right ventricle

**Fig. 2:**
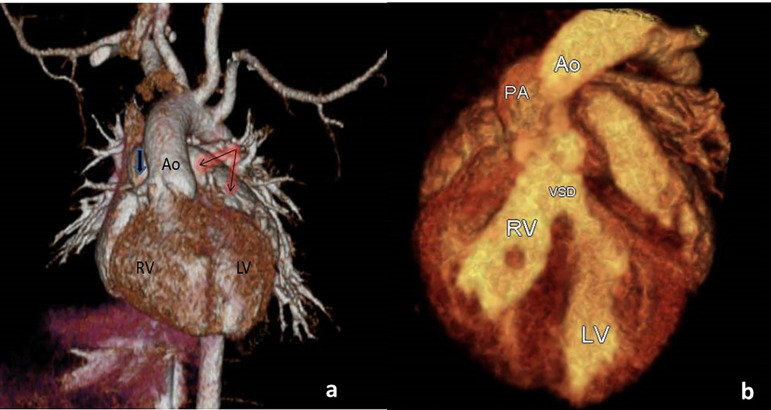
Three-dimensional computed tomography reconstruction image (a) representing the pulmonary trunk (bold arrow), aorta (Ao), and left juxtaposition of both atrial appendages (red shaded arrows); volume-rendering computed tomography image elaborating the anatomical relations in DORV (b). LV=left ventricle; PA=pulmonary artery; RV=right ventricle; VSD=ventricular septal defect

A 15-day-old newborn male was referred to our clinic for further evaluation of central cyanosis and previously detected heart murmur. Prenatal history was unremarkable with uneventful full term labour. During the initial physical examination, the baby was slightly cyanotic (SpO_2_: 88-90%) and the cardiac auscultation revealed a grade II systolic murmur at the upper left sternal border. Four extremity pulses were regular with approximate blood pressure results. The electrocardiogram was consistent with possible right ventricular hypertrophy and the chest X-ray demonstrated cardiomegaly due to enlarged right cavities, flat pulmonary trunk segment, and diminished pulmonary vascular markings. The transthoracic echocardiography revealed situs solitus, levocardia with normal pulmonary venous drainage, and atrioventricular concordance. Ventriculo-arterial relations were supplied by both the entire pulmonary trunk emerging from the right ventricle along with more than 50% of the aorta. DORV with anterior and left-sided aorta was evidenced (Figures 1A and 1B). Secundum type atrial septal defect of 10 mm in two-dimensional images maintaining left to right shunt, subaortic VSD approximately 9 mm in diameter with mostly left to right bidirectional flow, and right ventricular outflow tract obstruction originating from the subvalvular region generating a transpulmonic gradient of 50 mmHg were also noted. Both the left and right ventricular systolic functions were evaluated as preserved. There was no obstruction of the aortic arch and the pericardium had a normal appearance. For more detailed evaluation of the cardiac anatomy, cardiac computed tomography was performed (Figures 2A and 2B). The final diagnosis was DORV with situs solitus, D-loop ventricles, and L-position of the aorta {S,D,L}, left aortic arch associated with a large subaortic VSD, and pulmonary stenosis. Although the weight gain of the patient was sufficient with an uneventful follow-up, decremental course of the systemic saturation clarified the necessity of a surgical intervention at the age of six months. Surgery was performed via standard median sternotomy, and ascending aortic + bicaval cannulations were initiated afterwards. Anatomical relationship between the great vessels and intracardiac defects was coherent with the echocardiography. Additionally, juxtaposition of the right atrial appendage to the left and the sinus node artery passing through the anterior aspect of the right ventricular epicardium were also noticed perioperatively. DORV repair, including the Rastelli procedure (VSD closure with porcine pericardium, right ventricle-pulmonary trunk conduit: 14 mm Contegra® Medtronic, Inc. Medtronic Parkway Minneapolis, United States of America) and cessation of the antegrade flow by over-and-over suture technique, was performed. The postoperative course was uneventful, and the patient was discharged from the hospital on the postoperative 10^th^ day. The proceeding follow-ups were free of problems.

## DISCUSSION

DORV is defined as a subgroup of congenital heart defects in which both aorta and pulmonary trunk arise entirely or predominantly from the right ventricle. Ventriculo-arterial association is mostly defined as an aorta that overrides the septal crest by more than 50% and, therefore, lies principally over the right ventricle. The great arteries can be positioned in different combinations with each other, and the VSD can be located anywhere beneath those vessels. The most common form is being either normal (the pulmonary trunk is located anteriorly to the left and the aorta is posteriorly to the right) or having a side-by-side relation, for the great arteries, and a subaortic location, for the VSD ^[3,4]^. The most prevalent anatomic arrangement observed in DORV is the combination of normally committed great arteries with a subaortic VSD and a subpulmonary stenosis. In a large study with 393 DORV patients, Bradley et al. ^[1]^ reported their ventriculo-arterial relationships as concordant (42%), the most common, and L-malposed (8%), the least common. As previously mentioned, while levo-position of the great arteries in the concept of DORV is already a rare anomaly, the juxtaposition of both atrial appendages is seen even more rarely. Although some reports demonstrate their association with different other anomalies, there are only a few case reports of detection of both anomalies in the same patient ^[2,5,6]^. Our case report is important due to the rarity of this anatomical type of DORV.

The anatomic varieties found in DORV may result in diverse clinical discoveries and require different therapeutic approaches. The course after birth is determined mainly by the location of the VSD in relation to the great arteries as well as the presence or absence of outflow tract obstruction. The physiology of DORV displays similarity to the tetralogy of Fallot in case of subvalvular pulmonary stenosis resulting in cyanosis, as seen in the example of our case. The patient initially presented without severe cyanosis due to the subaortic VSD and gradually worsened owing to the progressive nature of his pulmonary stenosis.

As the anatomy of DORV patients are complicated with different additional pathologies, echocardiography is an indispensable tool on the way to diagnosis, providing the fact that the surgical management of this lesion mostly depends on the anatomic features. Additional diagnostic imaging work-up including computed tomography and magnetic resonance imaging would be beneficial for further evaluation of morphology before surgical intervention.

Due to the diversity in anatomy, surgical repair techniques should also be tailored to the individual. For patients with subaortic or doubly committed VSDs, without right ventricular outflow tract obstruction, the usual repair is an intraventricular tunnel from the VSD to the aorta. In case of right ventricular outflow tract obstruction, augmentation of the right ventricular outflow or conduit placement from the right ventricle to the pulmonary trunk would be necessary. In a literature review, DORV with L-position of the great arteries was usually corrected by constructing an intraventricular tunnel between the left ventricle and the aorta, removing the subpulmonary conal musculature, and enlarging the right ventricular outflow tract with a patch ^[1,7]^. In agreement with the latter statement, we selected the Rastelli procedure as our choice of surgical technique in this case to obtain the optimal anatomical repair, which resulted in an uneventful early and late postoperative course. For the DORV+{S,D,L} anatomy, the difficulty of intracardiac tunneling or existing ventricular hypoplasia may lead to a single ventricle repair. Coexisting atrial juxtaposition may complicate closure of the VSD through atriotomy, as observed in our case, so the closure was performed with an infundibular incision. Although juxtaposition has affected the VSD closure technique, it was still possible a biventricular repair.

In conclusion, as DORV+{S,D,L} associated with atrial juxtaposition is a rare condition, morphology should be fully evaluated and appropriate surgical approach should be performed with both echocardiographic and radiological supports.

**Table:** 

Authors' roles & responsibilities	
BÇ	Substantial contributions to the conception or design of the work; the acquisition or analysis of data for the work; revising the work critically for important intellectual content; final approval of the version to be published
EÖ	Agreement to be accountable for all aspects of the work in ensuring that issues related to the integrity of any part of the work are appropriately investigated and resolved; final approval of the version to be published
OY	Agreement to be accountable for all aspects of the work in ensuring that issues related to the integrity of any part of the work are appropriately investigated and resolved; final approval of the version to be published
SH	Agreement to be accountable for all aspects of the work in ensuring that issues related to the integrity of any part of the work are appropriately investigated and resolved; final approval of the version to be published
AG	Agreement to be accountable for all aspects of the work in ensuring that issues related to the integrity of any part of the work are appropriately investigated and resolved; final approval of the version to be published
